# Impact of Temperature and Substrate Type on the Optical and Structural Properties of AlN Epilayers: A Cross-Sectional Analysis Using Advanced Characterization Techniques

**DOI:** 10.3390/molecules29225249

**Published:** 2024-11-06

**Authors:** Wenwang Wei, Yi Peng, Yuefang Hu, Xiuning Xu, Quanwen Xie

**Affiliations:** 1 Guangxi Key Laboratory of Calcium Carbonate Resources Comprehensive Utilization, College of Materials and Chemical Engineering, Hezhou University, Hezhou 542899, China; 2 College of Electric Power Engineering, Guangxi Vocational College of Water Resources and Electric Power, Nanning 530023, China

**Keywords:** AlN, cross-sectional properties, temperature, warpage, Raman spectra

## Abstract

AlN, with its ultra-wide bandgap, is highly attractive for modern applications in deep ultraviolet light-emitting diodes and electronic devices. In this study, the surface and cross-sectional properties of AlN films grown on flat and nano-patterned sapphire substrates are characterized by a variety of techniques, including photoluminescence spectroscopy, high-resolution X-ray diffraction, X-ray photoelectron spectroscopy, ultraviolet photoelectron spectroscopy, and Raman spectroscopy. The results indicate that different sapphire substrates have minimal impact on the photoluminescence spectrum of the epitaxial films. As the temperature increased, the radius of curvature of the AlN films increased, while the warpage decreased. The AlN films grown on nano-patterned substrates exhibited superior quality with less surface oxidation. During the growth of AlN thin films on different types of substrates, slight shifts in the energy bands occurred due to differences in the introduction of carbon-related impurities and intrinsic defects. The Raman shift and full width at half maximum (FWHM) of the E_2_(low), A_1_(TO), E_2_(high), E_1_(TO), and E_1_(LO) phonon modes for the cross-sectional AlN films varied with the depth and temperature. The stress state within the film was precisely determined with specific depths and temperatures. The FWHM of the E_2_(high) phonon mode suggests that the films grown on nano-patterned substrates exhibited better crystalline quality.

## 1. Introduction

Owing to its ultra-wide bandgap and exceptional properties, such as high thermal conductivity and its high breakdown field strength, aluminum nitride (AlN) has emerged as a promising material for various high-performance applications, particularly in deep ultraviolet light-emitting diodes (DUV-LEDs) and electronic devices. Furthermore, its unique attributes, including its transparency in the UV region and thermal stability, make it an ideal candidate for substrate material in high-power and high-frequency electronic devices as well as for UV and DUV LEDs and laser diodes [1,2,3,4,5]. Despite these advantages, the performance of AlN in device applications relies on the quality of its epitaxial layers, which are affected by factors such as the growth temperature, substrate laser diode type, and the presence of impurities and defects.

To achieve high-quality AlN films, various growth techniques have been explored. Among them, metal-organic chemical vapor deposition (MOCVD) and physical vapor transport (PVT) are recognized as highly effective for the fabrication of single AlN crystals [5,6,7,8,9,10]. Additionally, AlN crystals are an ideal substrate material for the epitaxial growth of group III nitrides, which can resolve the issues of large lattice mismatches and large thermal mismatches associated with Si, sapphire, and SiC substrates [11]. However, it is still challenging to further reduce the dislocation density and improve the overall structural and optical properties of AlN epilayers. In this context, examining the effects of the substrate and temperature on the growth process is of paramount importance. Nano-patterned substrates have shown promise in enhancing the quality of AlN films by reducing the dislocation density and managing strain [12,13,14].

Xie et al. [15] demonstrated that achieving a critical balance between reducing the area ratio of the coalescence zone and decreasing the coalescence thickness is essential in pattern design. By growing AlN films on nano-patterned sapphire substrates (NPSSs), they achieved high-quality AlN templates and improved stress status and verified the feasibility of fabricating high-performance DUV-LEDs based on the period size effect on NPSSs. Wang et al. [16] investigated the coalescence, stress evolution, and dislocation annihilation mechanisms in the AlN layer, suggesting that the threading dislocations (TDs) primarily originated from the boundary between misaligned crystallites and the c-plane AlN, as well as from the coalescence of two adjacent c-plane AlN crystals, rather than from the interface between the sapphire and AlN. Wang et al. [17] also emphasized the use of nano-patterned AlN/sapphire templates to achieve group III nitride heteroepitaxial films with bulk-class quality by controlling the discretization and coalescence of AlN columns. Additionally, Xu et al. [18] reviewed advances in epitaxial growth and defect control of AlN on sapphire substrates. By employing techniques such as nitridation pretreatment of sapphire to reduce the tilt of AlN grains, forming TD loops through three-dimensional/two-dimensional (3D-2D) alternate growth, bending and terminating TDs at the sidewall of voids using the image force on NPSSs, and promoting the climbing and meeting of TDs by extrinsic supersaturated vacancies, AlN films with a low threading dislocation density (TDD) were obtained on sapphire substrates, laying a solid foundation for the realization of high-performance AlGaN-based DUV-LEDs. Kneissl et al. [19] underscored the importance of high-quality AlN templates in enhancing device performance and provided guidance for the future development of III-nitride DUV-LED technology. Zhang et al. [20] explored the relationship between the dislocation density and stress during the growth of AlN via MOCVD. They found that by controlling the size and density of crystal islands at the end of 3D growth, the tensile stress generated during epitaxy could be effectively reduced. Furthermore, the results indicated that the three-step growth method was more effective in reducing dislocations and tensile stress than the method using patterned sapphire substrates (PSSs) instead of flat substrates. In a study conducted by Wei et al. [21], the root mean square (RMS) surface roughness of the S1 and S2 substrates was found to be 2.32 and 2.36 nm, respectively, in a 5 × 5 μm^2^ region. Spectroscopic ellipsometry fitting at 300 K yielded bandgaps of 6.16 eV and 6.06 eV for the S1 and S2 samples, respectively. The authors investigated the effects of temperature on the stress and optical properties of AlN thin films, further revealing the influence of temperature on the material’s optical properties. However, the critical thickness and temperature at which the cross-sectional temperature-dependent Raman spectroscopy and stress transformation of AlN films occur remain largely unexplored. A comprehensive understanding of the surface and cross-sectional properties is crucial for the fabrication of group III nitride-based optoelectronic devices.

In this study, we systematically examine the effects of the temperature and substrate type on the optical and structural properties of AlN epitaxial layers. Using advanced characterization techniques, such as photoluminescence (PL) spectroscopy, high-resolution X-ray diffraction (HRXRD), X-ray photoelectron spectroscopy (XPS), ultraviolet photoelectron spectroscopy (UPS), and Raman spectroscopy, the AlN thin films grown on both flat substrates and NPSSs are thoroughly analyzed. Overall, our findings provide valuable insights into temperature-dependent warpage, the impact of substrate nano-patterning on film quality, and depth- and temperature-dependent cross-sectional Raman spectra characteristics. These insights are crucial for optimizing the growth process, enhancing device performance, and managing strain effectively. Additionally, they offer new perspectives on transport mechanisms and the unique properties of AlN.

## 2. Results and Discussion

**Figure 1:**
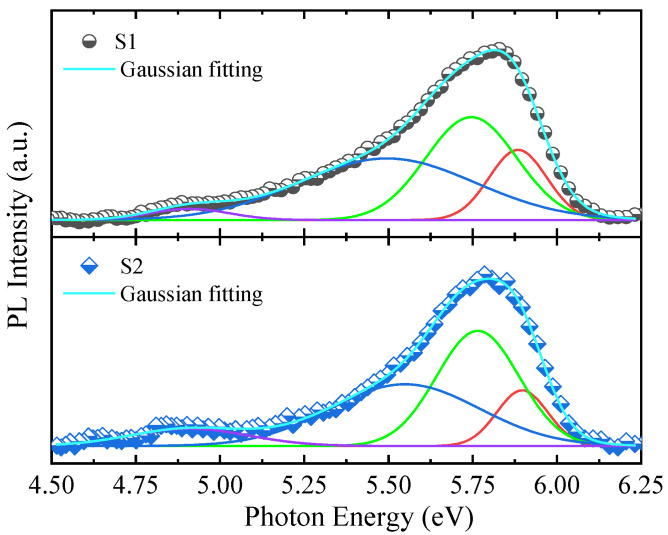
PL spectra of samples S1 and S2 excited by a 193 nm laser.

Figure 1 shows a typical PL spectrum of the AlN film excited by a 193 nm laser. The dominant fluorescence emission peak of AlN appeared at approximately 5.75 eV. Gaussian fitting revealed additional sub-peaks at 4.91, 5.50, and 5.88 eV. The 4.91 eV band is associated with radiative recombination between the conduction band and the level aluminum vacancy-related defect. Mi et al. [22] also observed an additional broad Al vacancy related peak at 4.86 eV, which is consistent with the calculated transition energy between conduction band and the single positively charged Al vacancy state. The peaks at 5.75 and 5.88 eV were attributed to the free exciton recombination and its associated longitudinal optical (LO) phonon replica, with an LO phonon energy of 130 meV in AlN. The splitting of the emission band may be due to the Al-N bond anisotropy. The bandgap of AlN was indeed over 6.0 eV, with values of 6.16 eV and 6.06 eV obtained through spectroscopic ellipsometry fitting for samples S1 and S2, respectively. The discrepancy between the PL emission peaks and the bandgap energy suggests that the observed PL peaks may not have been due to near-bandgap transitions but could be related to other processes. In semiconductor physics, emission peaks below the bandgap may originate from radiative recombination involving defects. Defects can introduce energy levels within the bandgap, allowing for recombination processes which emit photons at lower energies. The presence of native point defect recombination centers partially accounts for the reduced DUV emission efficiency in AlN [23]. The PL spectra showed minor dependence on the dislocation densities and substrate type.

**Figure 2:**
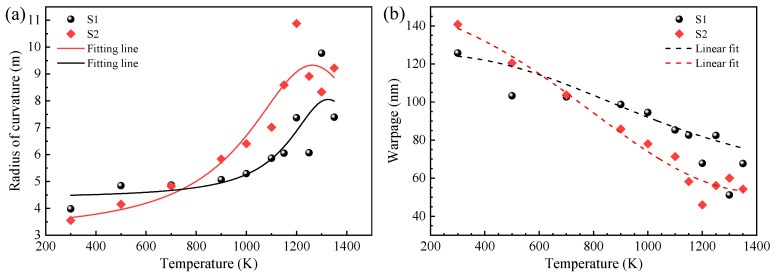
(**a**) Variation in the radius of curvature versus temperature. (**b**) Variation in the warpage against temperature for the AlN samples.

LED devices generate a certain amount of heat in their operating state, which affects their performance and lifespan. The temperature-induced changes in the radius of curvature for two LED samples, S1 and S2, are depicted in Figure 2a, spanning a temperature range from 300 K to 1350 K. The results reveal that the radius of curvature for sample S1 experienced a gradual increase with rising temperatures, followed by a sharp rise at temperatures surpassing 1000 K. Interestingly, there was a reversal, with the radius of curvature beginning to decrease at 1250 K. Sample S2 exhibited a parallel trend, albeit with a more pronounced rate of change. These discrepancies between the two samples can be attributed to the distinct substrate materials used. The implementation of NPSS is noted for its efficacy in mitigating the dislocation density within the films and alleviating residual internal stress. Figure 2b illustrates the temperature-dependent warpage behavior of the AlN samples. It can be observed that the warpage of both samples decreased as the temperature increased. This behavior is particularly intriguing when considering the thermal expansion coefficients of AlN and sapphire in the direction perpendicular to the c-axis, which were 4.2 × 10^−6^ K^−1^ and 7.5 × 10^−6^ K^−1^, respectively. The thermal stress experienced by the AlN layers due to the mismatch in thermal expansion coefficients with the sapphire substrate can lead to deformation and potential delamination, which are critical factors affecting the reliability and longevity of LED devices. The AlN epitaxial layers under thermal stress were found to be under tensile stress, a finding corroborated by the variable-temperature cross-sectional Raman spectra presented in the following discussion. Furthermore, the decrease in warpage with increasing temperatures suggests that the internal stresses within the samples were relieved as the temperature rose, which could be due to the reduction in the modulus of elasticity of the materials at higher temperatures. However, the eventual decrease in the radius of curvature for sample S1 at extremely high temperatures (1250 K) indicates that other thermal degradation processes may work.

**Figure 3:**
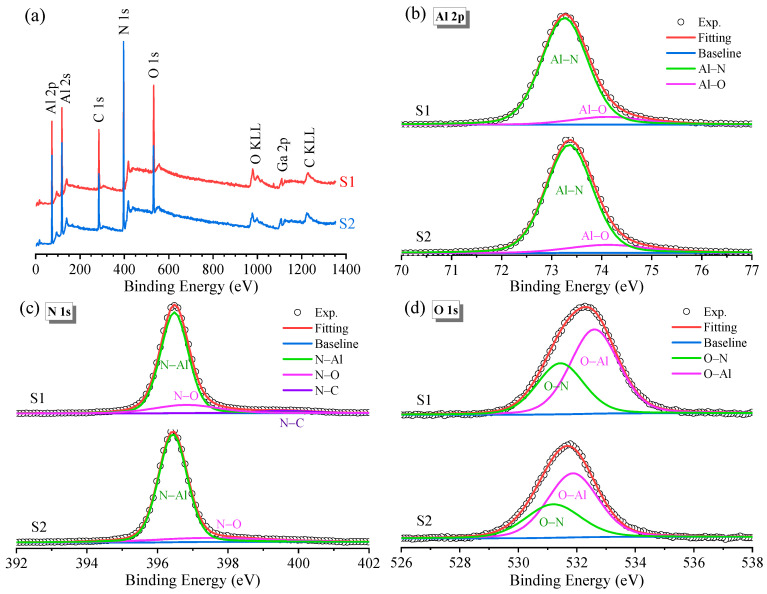
(**a**) XPS survey spectra, with high-resolution XPS spectra of Al 2p (**b**), N 1s (**c**), and O 1s (**d**) for the AlN samples.

Figure 3a displays the XPS survey spectra for samples S1 and S2. The XPS survey spectra of the AlN samples revealed the presence of Al, N, C, and O. Due to incomplete surface cleaning, a large amount of carbon was detected, likely resulting from exposure to the atmosphere or impurities adsorbed on the sample surface. This spectrum showed protruding oxygen and carbon peaks, with the oxygen peak being more pronounced in sample S1, indicating greater surface oxidation. This can be attributed to the differences in the surface area or surface energy of the substrates, with the flat substrate (S1) potentially providing more sites for oxygen adsorption. Surface oxygen can originate from two primary sources: growth-induced oxidation and doping. Firstly, AlN materials are prone to oxidation due to their inherent oxidation characteristics and the high reactivity of aluminum. Secondly, oxygen can also be introduced during the growth process, particularly due to the rapid reaction of trimethylaluminum precursors with oxygen in the gas stream. Moreover, a signal of about 1100 eV was observed in the XPS survey spectrum, which is typical for the Ga 2p core level. We suspect that the Ga impurity came from the vacuum chamber of MOCVD system not being completely cleaned, in which Ga may have become a residual impurity after GaN or AlGaN film growth in the same system. Chen et al. [24] also reported that there always exist Ga, O, and C contaminations in AlN films.

The oxygen impurities on the surface of AlN films can react with aluminum and nitrogen, affecting the properties of the films. The high-resolution XPS scans of Al 2p, N 1s, and O 1s could be deconvoluted into two or three sub-peaks, and the fitting parameters for these sub-peaks are presented in Table 1. In Figure 3b, the Al 2p core level spectrum is deconvoluted into two subpeaks corresponding to Al-N and Al-O bonds, centered at 73.26 and 74.12 eV for sample S1, respectively. The binding energy corresponding to the Al-N peak in the S2 sample was 90 meV larger than that in sample S1, while the Al-O bond energy remained the same. Due to the higher electronegativity of oxygen, the peak shifted to a higher binding energy position when aluminum bound to oxygen. Thus, the peak at 74.12 eV was assigned to the Al-O bond. The N 1s spectrum in Figure 3c can also be decomposed into two components, corresponding to the N-Al and N-O bonds. For sample S1, a small peak at 399.59 eV was attributed to the N-C bond, which arose from contamination due to double bonds formed with carbon. In Figure 3d, the O 1s spectrum is deconvoluted into two major peaks centered at 531.44 and 532.60 eV, which are attributed to the O-N and O-Al bonds, respectively. The binding energy for forming the same chemical bond can vary depending on experimental factors such as charge effects, electric fields, hybridization, and the ambient charge density [25].

A thin oxide layer was formed on the AlN surface, which could protect AlN materials from further oxidation to a certain extent. In the process of forming oxides on the surface of AlN films, oxygen replaced the nitrogen atoms originally bound to Al, resulting in the formation of oxides and the accumulation of Al on the surface. Once the deposited film was exposed to air, the outer surface of AlN would oxidize to form Al_2_O_3_ until the oxidation layer densification or passivation of the AlN material reaches equilibrium. However, this layer can also have a detrimental effect on the electrical and optical properties of AlN films [26]. The thickness of the oxide layer on the surface *d_XPS_* (nm) was estimated from the intensity ratio of the Al 2p alumina oxide peak to the alumina nitride peak (*I_o_*/*I_m_*) using the following equation:

(1) $$d_{XPS}\left(nm\right)=\lambda_{0}\sin\theta \ln\left(\frac{N_{m}\lambda_{m}I_{o}}{N_{o}\lambda_{o}I_{m}}+1\right)$$

where the ratio of the volume densities of aluminum atoms in nitrides to oxides is *N_m_*/*N_o_* = 1.6. *λ_o_* (2.92 nm) and *λ_m_* (2.39 nm) are the effective attenuation lengths of alumina and aluminum nitride, respectively [27]. The angle *θ* between the X-ray source and the axis of the electron lens was 45°. The thicknesses of the surface oxide layers for samples S1 and S2 were 3.89 and 3.14 nm, respectively. The results indicate that the surface of AlN epitaxial films grown on a nano-patterned substrate is less susceptible to oxidation.

**Figure 4:**
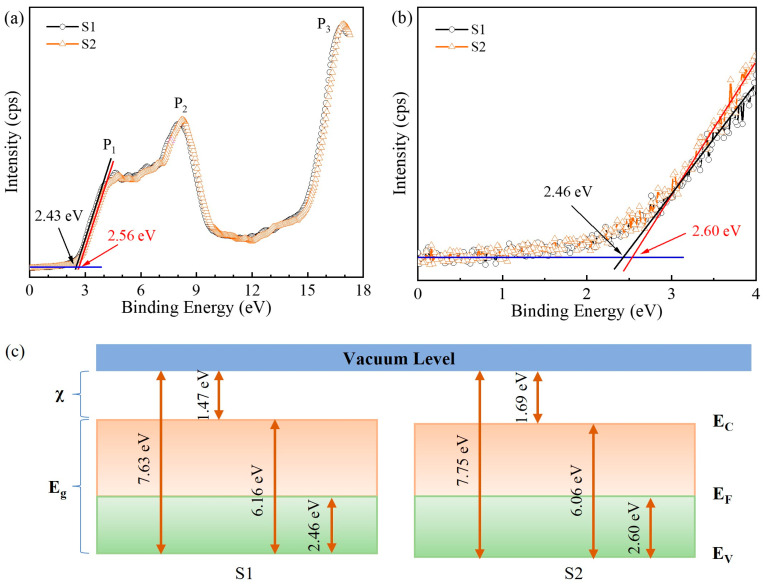
(**a**) XPS valence band spectra, (**b**) UPS valence band spectra, and (**c**) band structure of the AlN/CFSS and AlN/NPSS samples.

The XPS valence band spectrum of the AlN samples is presented in Figure 4a. The three peaks near 4.5, 8.2, and 16.8 eV are labeled P_1_, P_2_, and P_3_, respectively. The valence band maxima (VBM) for the S1 and S2 samples were observed at 2.43 and 2.56 eV, respectively. This variation is attributed to the different internal stresses induced by different substrates. Additionally, carbon-related impurities and intrinsic defects might contribute to this observed difference. The surface potential barriers, defined as the energy difference between the conduction band minimum and the Fermi level, were calculated to be 3.73 eV and 3.50 eV for the S1 and S2 samples, respectively. To further elucidate the energy band alignment and analyze the effect of NPSS, UPS measurements were performed, and the results are shown in Figure 4b. The VBM values obtained from the UPS spectra were 2.46 eV and 2.60 eV for samples S1 and S2, respectively. The higher VBM values from the UPS spectra mainly stemmed from the greater surface detection sensitivity of the UPS system. For semiconductor materials, the electron affinity (*χ*), which is the minimum energy required for electrons to reach the vacuum level from the conduction band minimum, is an important parameter influenced by the surface morphology. The electron affinity can be determined as follows:

(2) $$\chi =hv-W-E_{g}$$

where *hν* is the energy of the incident photons, *W* is the spectral width from the VBM to the low energy cut-off, and *E_g_* is the bandgap of the material [28]. The electron affinity was determined to be 1.47 eV and 1.69 eV for samples S1 and S2, respectively, as shown in Figure 4c. The ionization energy of the electrons was calculated to be 7.63 eV and 7.75 eV for the S1 and S2 films, respectively. The observed changes in electronic properties possibly originated from strain relaxation [29]. However, Liang et al. concluded that the electron affinity and ionization energy increased with the increase in film thickness and oxygen content, with the oxygen content playing a dominant role [30].

**Figure 5:**
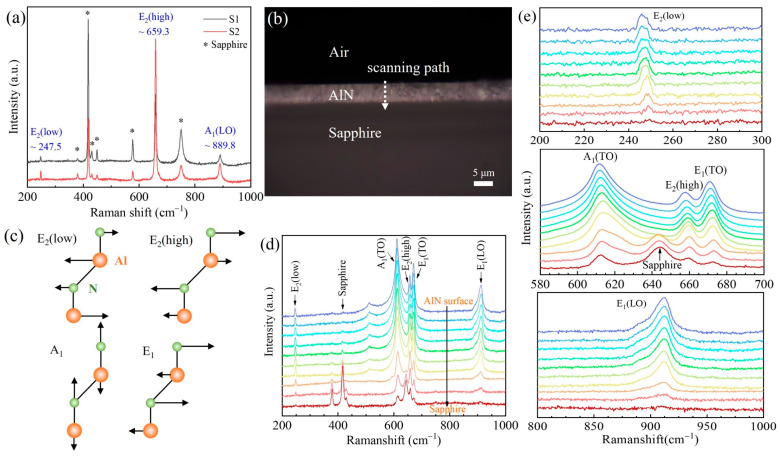
(**a**) Surface Raman spectra of AlN epilayers. (**b**) Schematic diagram of cross-sectional Raman spectroscopy test. (**c**) Atomic vibration modes in AlN with the *c* axis oriented upward. (**d**) Variation in the cross-sectional Raman spectrum from the AlN surface to the sapphire substrate. (**e**) Partial magnification of the peaks corresponding to the E_2_(low), A_1_(TO), E_2_(high), E_1_(TO), and E_1_(LO) phonon modes.

The surface Raman spectra were recorded with the laser light incident normal to the surface of the AlN epilayers, as shown in Figure 5a. The frequency shift and linewidth of the E_2_(high) mode were used to evaluate the crystalline quality and residual stress of the epitaxial film. The Raman shifts of the E_2_(high) mode were larger than the stress-free frequency of AlN (ω_0_ = 657.4 cm^−1^) [31], indicating that the AlN epilayers were under residual compressive stress. In the backscattering geometry, the E_2_ and A_1_(LO) modes are allowed in AlN with the *c* axis oriented upward, whereas the A_1_(TO) and E_1_(TO) phonon modes are forbidden. A schematic of the cross-sectional Raman spectroscopy test for sample S2 is presented in Figure 5b, where the cross-sectional thickness and morphology are clearly visible.

Based on group theory, Figure 5c illustrates the various atomic vibrational modes in hexagonal AlN with the *c* axis oriented upward. The E_2_ modes were Raman-active, while the A_1_ and E_1_ modes were both Raman- and infrared-active. In the surface Raman spectra, the E_2_(high) peaks had the strongest intensity. However, in the cross-sectional Raman spectra, the intensity of the A_1_(TO) peaks was the highest. This is consistent with the selection rules for semiconductor materials with wurtzite structures. The phonon mode was more easily excited when the laser beam was incident perpendicular to the direction of phonon mode vibration.

It is evident in Figure 5d that there were five phonon modes in the cross-sectional Raman spectrum of AlN: E_2_(low), A_1_(TO), E_2_(high), E_1_(TO), and E_1_(LO). The peaks of these phonon modes exhibited a blueshift as the probing depth increased from the surface toward the substrate, as shown in the partial magnified spectra in Figure 5e. The E_2_ mode corresponded to atomic vibrations in the *c* plane, with the E_2_(high) mode being extremely sensitive to the lattice strain in the *c* plane. The shift in the E_2_(high) mode reflects the presence of different strains at various cross-sections from the sample surface to the substrate. The blueshift of the E_2_(high) mode indicates that the residual compressive stress increased with the depth, which is in agreement with the expected results. At the end of growth, the sapphire substrate shrank faster than the AlN epilayer upon cooling, which is ascribed to the different thermal expansion coefficients of AlN and the substrate. This resulted in the generation of compressive strain in the AlN epilayer, which was greater near the interface between the AlN film and the sapphire substrate.

**Figure 6:**
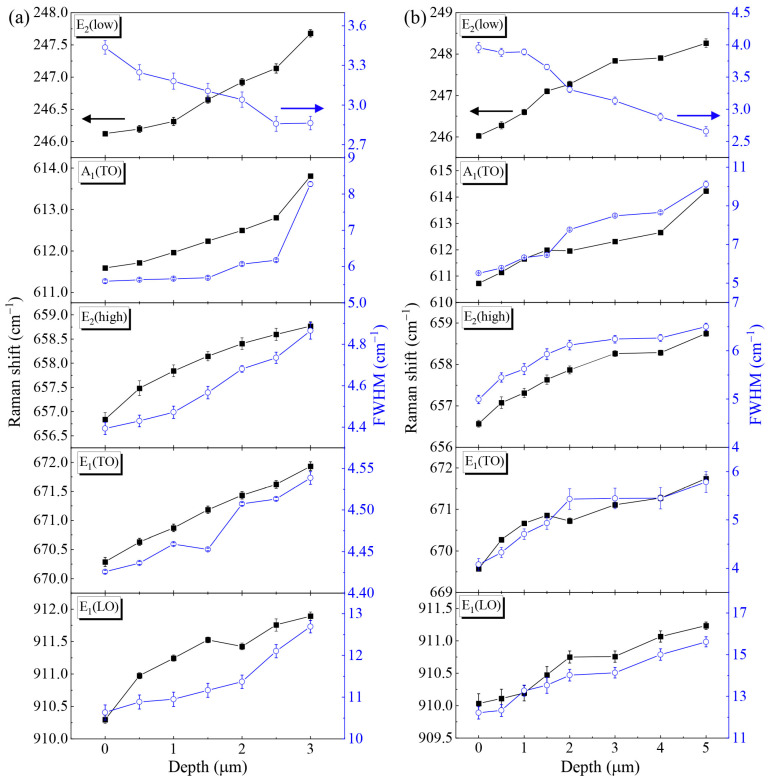
Variation in the E_2_(low), A_1_(TO), E_2_(high), E_1_(TO), and E_1_(LO) phonon modes with depth for (**a**) sample S1 and (**b**) sample S2.

Figure 6 presents the Raman shift and full width at half maximum (FWHM) as functions of the depth for samples S1 and S2. For sample S1, the E_2_(low), A_1_(TO), E_2_(high), E_1_(TO), and E_1_(LO) modes were continuously blue-shifted with the increase in the laser incidence depth within the thickness range of AlN. The variation in the Raman shift was generally within 2 cm^−1^. It is worth noting that the FWHM of sample S2 was larger. For this, there are several reasons for our analysis. (1) The nanopatterned substrate provides more channels for stress release. The AlN film grown on the NPSS can gradually release stress during the growth process due to the existence of nanopores. This stress release may result in an increase in defects and distortions in the crystal structure, thereby increasing the broadening of the Raman peak. (2) The nanopatterned substrate can more effectively reduce the stress caused by lattice mismatching and reduce the dislocation density. Lower stress and dislocation densities can reduce crystal distortion and theoretically reduce the broadening of the Raman peak. However, if the nanopatterned substrate introduces additional surface or interface defects, these defects may increase the non-uniformity of Raman scattering, resulting in an increase in the FWHM. (3) The nanopatterned substrate may increase the light scattering and coupling effects, which may affect the measurement results of Raman spectra and cause broadening of the peak position.

Regarding the FWHM, the FWHM of the E_2_(high) peak in AlN serves as a useful indicator of crystallinity, with the best crystalline quality observed near the sample surface. The variation trends of the Raman shifts and FWHM for each phonon mode of sample S2 were basically consistent with those of sample S1. However, for the E_2_(high) mode, the Raman shift in sample S2 was smaller than that in S1, indicating that sample S2 experienced less compressive stress in the AlN epilayer. Notably, the Raman shifts at the surface for both samples were below 657.4 cm^−1^, suggesting the presence of tensile stress.

**Figure 7:**
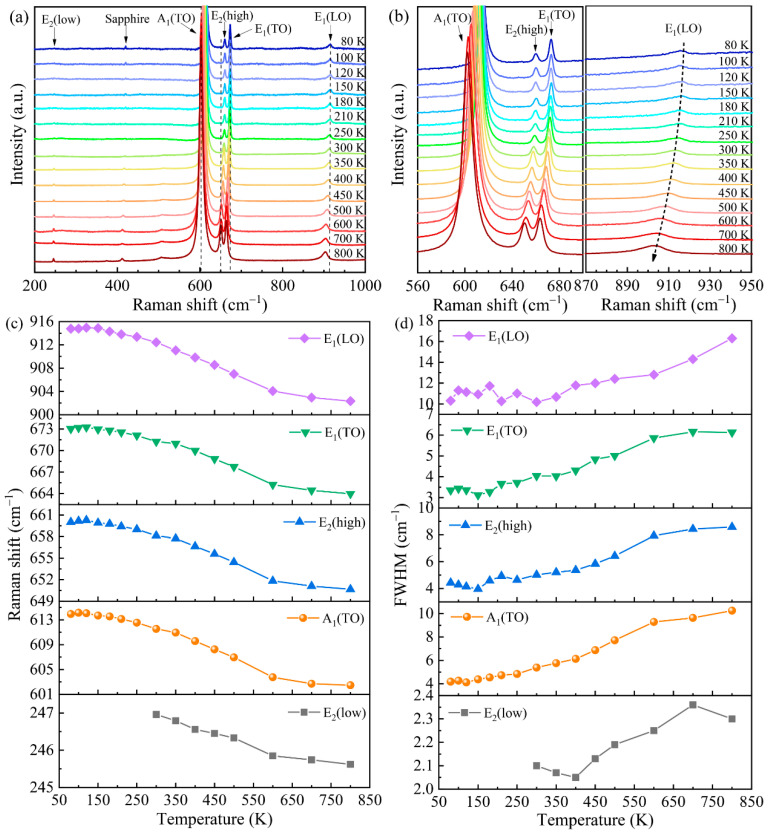
(**a**) Cross-sectional variable-temperature Raman spectra of sample S2. (**b**) Partial magnification of (**a**). (**c**) Temperature-dependent Raman shift of sample S2. (**d**) FWHM of sample S2 as a function of temperature from 80 to 800 K.

To examine the effect of temperature on the cross-sectional Raman spectra, the cross-sections of the S2 samples were characterized in the temperature range of 80–800 K, and the results are shown in Figure 7a. At low temperatures, the peak of E_2_(low) was rather weak. It is evident in Figure 7b that the peaks of the A_1_(TO), E_2_(high), E_1_(TO), and E_1_(LO) phonon modes exhibited significant red shifts as the temperature rose from 80 to 800 K. This red shift can be attributed to two factors: (1) the thermal expansion of the lattice, which changed nonlinearly with the temperature and was anisotropic, with the expansion along the *a* axis being larger than that along the *c* axis, and (2) the anharmonic effect of two-phonon interactions, which caused a red shift with the increase in temperature. Figure 7c,d shows the Raman shifts and FWHMs of the five phonon modes of AlN as functions of the temperature, respectively. The variation trends for the frequency shift and linewidth with temperature were the same for the five phonon modes. At 300 K, the Raman shift was determined to be 658.1 cm^−1^ for the AlN epilayer, which is slightly larger than the stress-free value of 657.4 cm^−1^, indicating the presence of small compressive stress. In addition, the Raman linewidth of the E_2_(high) mode at 300 K was 5.02 cm^−1^, which is comparable to the previously reported value of 3.3–6.5 cm^−1^ for AlN bulk single crystals [32]. The FWHM of the E_2_(high) mode varied between 3.9 and 8.6 cm^−1^ over the temperature range of 80–800 K. This variation in the FWHM with temperature is associated with point defect scattering. Generally, broadening of the Raman linewidth in a crystal film results from a reduction in the phonon lifetime due to scattering.

**Figure 8:**
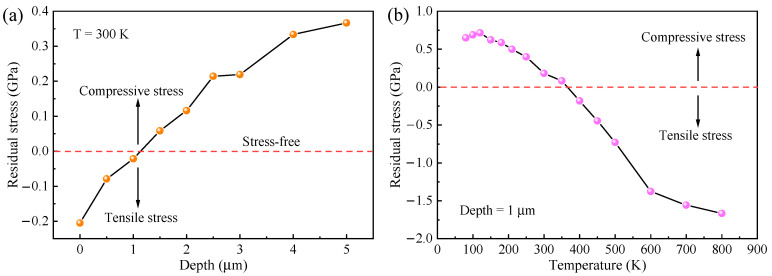
(**a**) Relationship between the residual stress and depth. (**b**) Temperature dependence of the residual stress.

The stress (*σ*) can be evaluated as follows:

(3) $$\sigma =\frac{\omega -\omega_{0}}{\kappa }$$

where *ω* is the experimental peak position and *ω*_0_ = 657.4 cm^−1^. The biaxial stress coefficient *κ* was 4.04 cm^−1^/GPa for the E_2_(high) mode of AlN [33]. The residual stress values as a function of the depth are depicted in Figure 8a. These values indicate that the residual tensile stress was present within a thickness of approximately 1 µm from the surface, while the remaining regions exhibited residual compressive stress. This observation is consistent with the previous results from the surface Raman spectrum. The temperature dependence of the residual stress is illustrated in Figure 8b. As the temperature increased, the residual stress in the AlN film transformed from compressive to tensile stress at 400 K. Typical sources of such residual stress include differences in thermal expansion coefficients, lattice mismatches in heterogeneous epitaxial growth, and the presence of grain boundaries, defects, and impurities [34].

## 3. Materials and Methods

The AlN epilayers were grown via metal-organic chemical vapor deposition (MOCVD) on 2 inch conventional flat sapphire substrate (CFSS) and NPSS, (CRYSCORE, Wuhan, China), labeled as S1 and S2, respectively, along the [0001] direction. The nano-patterned substrate featured 400 nm deep truncated cone patterns with a top diameter of 550 nm and a bottom diameter of 650 nm. The pitch length was approximately 1000 nm. The sapphire substrates were coated with a ~15 nm AlN layer through physical vapor deposition (PVD).

For the MOCVD epitaxial growth, the precursors used were trimethyl-aluminum (TMAl) for Al and ammonia (NH_3_) (Nata Opto-Electronic Materials Co; LTD, Jiangsu, China) for N. H_2_ was used as the carrier gas. Firstly, the sapphire substrates with the PVD AlN nucleation layer were heated up to 1150 °C in ambient H_2_, followed by the growth of an initial AlN roughing layer. The temperature was then increased to 1250 °C to restore the surface and maintain 2D growth. The thickness of the AlN epitaxial layer for the S1 and S2 samples was 3 μm and 5 μm, respectively. This thickness selection was mainly based on the observation that, for the patterned substrate, an AlN epitaxial layer thickness of 2 μm was needed to completely merge the wedge-shaped holes.

The fluorescence performance was characterized using PL spectroscopy (HORIBA Jobin Yvon, Paris, France) equipped with a 193 nm laser as the excitation source. The variable temperature (300–1350 K) warpage of the AlN materials was determined using HRXRD (Malvern PANalytical, Alemlo, The Netherlands) with a Ge (220) four-crystal monochromator, utilizing Cu Kα1 radiation (1.5406 Å) with an angular resolution of approximately 12 arcsec. The samples were laser-cut into 1 inch wafers and placed on a DHS-1100 domed hot stage capable of reaching temperatures up to 1373 K. XPS (Thermo Fisher Scientific, Waltham, MA, USA) experiments were conducted using a monochromatic Al Kα (1486.6 eV) radiation source with a filament power of 72 W. The vacuum pressure in the analysis chamber was less than 5.0 × 10^−9^ mbar. The XPS survey spectra and high-resolution spectra were collected with pass energies of 100 and 50 eV, respectively. The binding energy calibration was performed using a C 1s (284.8 eV) spectrum from a surface contamination layer as the standard. The core-level peaks were fitted using XPSPEAK4.1 software (version 4.1) by employing a Voigt mixture of the Gauss–Lorentz function and Shirley background model. UPS (Thermo Fisher Scientific, Waltham, MA, USA) measurements were carried out using a He (I) (21.2 eV) radiation source with a voltage of 15 kV and a beam current of 15 mA. Micro-Raman spectroscopy (HORIBA Jobin Yvon, Paris, France) was performed at room temperature and variable temperatures on both samples by using the backscattering geometry with a 532 nm He-Cd laser both normal to the surface and across the cross-section of the epilayers. Temperature-dependent Raman spectroscopy measurements were performed using a Linkam heating system.

## 4. Conclusions

In summary, AlN films were deposited on CFSS and NPSS via MOCVD. The surface and cross-sectional properties of these films were characterized by various techniques, including PL, HRXRD, XPS, UPS, and Raman spectroscopy. Using a 193 nm laser, the PL emission peak was obtained at 5.75 eV, and the sapphire substrate had a negligible effect on the PL spectrum. The PL spectra showed minor dependence on the dislocation densities and substrate type, indicating that both types of substrates did not significantly impact the PL properties of the AlN films. The radius of curvature and warpage measurements revealed that as the temperature increased from 300 to 1350 K, the radius of curvature of the AlN films increased, while the warpage decreased for both types of substrates. However, the nano-patterned substrate (S2) exhibited a greater rate of change in the radius of curvature with the temperature, suggesting that it might be more effective in reducing the dislocation density and releasing residual internal stress. The energy band alignment analysis revealed different electron affinity potentials for samples S1 and S2 (1.47 eV and 1.69 eV, respectively). The presence of carbon-related impurities and intrinsic defects might contribute to deviations in the energy band structure. The Raman shift and FWHM of the phonon modes indicated different levels of strain and crystalline quality for the films grown on the two substrates. The E_2_(high) phonon mode showed that the crystalline quality of S2 was superior, which was attributed to the modification effect of the NPSS on the epitaxial growth. The residual stress analysis indicated that the AlN film grown on the nano-patterned substrate (S2) had less compressive stress compared with the film grown on the flat substrate (S1). The stress transformation from compressive to tensile stress was observed in the AlN film as the temperature increased, which is crucial for device performance and lifetimes. The residual stress can be obtained by depth- and temperature-dependent cross-sectional Raman spectroscopic analysis, and the transformation of the AlN film stress can be realized, providing valuable insights for controlling the internal stress in film growth engineering.

## Figures and Tables

**Table 1:** Fitting parameters of the core-level peaks in Al 2p, N 1s, and O 1s spectra for the surfaces of the AlN films.

Core Level	Chemical Bond	S1 (AlN/CFSS)			S2 (AlN/NPSS)		
		Binding Energy (eV)	FWHM (eV)	Area (%)	Binding Energy (eV)	FWHM (eV)	Area (%)
Al 2p	Al−N	73.26	1.08	90	73.35	1.09	89
	Al−O	74.12	1.69	10	74.12	1.86	11
N1s	N−Al	396.48	0.93	81	396.44	0.96	88
	N−O	396.82	2.13	15	397.45	3.78	12
	N−C	399.59	1.91	4	—	—	—
O1s	O−N	531.44	1.88	37	531.20	2.22	36
	O−Al	532.60	1.95	63	531.87	2.04	64

## Data Availability

Data are contained within the article.
